# Von Willebrand Factor, ADAMTS13 and D-Dimer Are Correlated with Different Levels of Nephropathy in Type 1 Diabetes Mellitus

**DOI:** 10.1371/journal.pone.0132784

**Published:** 2015-07-13

**Authors:** Caroline Pereira Domingueti, Luci Maria S. Dusse, Rodrigo Bastos Fóscolo, Janice Sepúlveda Reis, Joyce Maria Annichino-Bizzacchi, Fernanda Loureiro de Andrade Orsi, Bruna de Moraes Mazetto, Maria das Graças Carvalho, Karina Braga Gomes, Ana Paula Fernandes

**Affiliations:** 1 Department of Clinical and Toxicological Analysis, Faculty of Pharmacy, Federal University of Minas Gerais, Belo Horizonte, Minas Gerais, Brazil; 2 Department of Medical Clinic, Faculty of Medicine, Federal University of Minas Gerais, Belo Horizonte, Minas Gerais, Brazil; 3 Department of Endocrinology and Metabolism, Institute of Education and Research of Santa Casa of Belo Horizonte, Belo Horizonte, Minas Gerais, Brazil; 4 Laboratory of Hemostasis, Hemocenter of Campinas, University of Campinas, São Paulo, Brazil; National Centre for Scientific Research “Demokritos”, GREECE

## Abstract

We have investigated whether von Willebrand factor, ADAMTS13 (a disintegrin and metalloproteinase with thrombospondin type 1 motif, member 13), and D-Dimer were associated with different levels of renal function in patients with type 1 diabetes. Patients were classified according to level of renal function through estimated glomerular filtration rate: ≥90 and <130mL/min/1,73m^2^, n=52 (control group), ≥60 and <90mL/min/1,73m^2^, n=29 (mild renal dysfunction group), <60mL/min/1,73m^2^, n=28 (severe renal dysfunction group); and through urinary albumin excretion: normoalbuminuria, microalbuminuria and macroalbuminuria. Von Willebrand factor, ADAMTS13, and D-Dimer plasma levels were determined by enzyme-linked immunosorbent assay. ADAMTS13 activity was determined by fluorescence resonance energy transfer assay. Von Willebrand factor levels were increased in patients with mild (P=0.001) and severe (P<0.001) renal dysfunction as compared to the control group. ADAMTS13 levels were also increased in mild (P=0.029) and severe (P=0.002) renal dysfunction groups in comparison to the control group, while ADAMTS13 activity was increased only in the severe renal dysfunction group as compared to the control group (P=0.006). No significant differences were observed among the groups regarding von Willebrand factor/ADAMTS13 ratio. ADAMTS13 activity/ADAMTS13 levels ratio was reduced in patients with mild (P=0.013) and severe (P=0.015) renal dysfunction as compared to the control group. D-Dimer levels were increased in patients with mild (P=0.006) and severe (P<0.001) renal dysfunction as compared to the control group; it was also higher in patients with severe renal dysfunction as compared to the mild renal dysfunction group (P=0.019). Similar results were found for albuminuria classification. Increased von Willebrand factor, ADAMTS13, and D-Dimer levels and decreased ADAMTS13 activity/ADAMTS13 levels ratio are associated with renal dysfunction in patients with type 1 diabetes, suggesting that endothelial dysfunction and hypercoagulability are associated with nephropathy in type 1 diabetes.

## Introduction

Diabetic nephropathy is the most common cause of end stage renal disease (ESRD) worldwide, contributing to approximately 45% of new cases [1, 2]. Early detection of nephropathy in diabetic patients is, therefore, essential to preventing or delaying the progression of renal disease and the development of cardiovascular complications [3].

Metabolic abnormalities resulting from chronic hyperglycemia and the associated inflammatory state in diabetic patients may lead to endothelial injury and consequent vascular complications. It has been suggested that endothelial dysfunction and hypercoagulability state are the earliest indicators of this process [4].

Von Willebrand factor (VWF) is a multimeric glycoprotein involved in primary hemostasis and in the coagulation process, acting as a carrier of factor VIII, which prevents its degradation by activated protein C. As VWF is released when endothelial cells are injured, it is an important biomarker of endothelial dysfunction [5]. VWF promotes platelet adhesion at vascular damage sites, where it mediates the progression of thrombus formation through specific interactions with subendothelial collagen and platelet receptors [6]. Elevated VWF has been found in patients with type 1 diabetes mellitus (DM1) and has been identified as a predictive biomarker of micro and macroangiopathy in these patients [7].

ADAMTS13 (a disintegrin and metalloproteinase with thrombospondin type 1 motif, member 13) is an enzyme responsible for the cleavage of large multimers of VWF, released into plasma by endothelial cells and platelets, and an imbalance between VWF and ADAMTS13 plasma levels may also contribute to the development of micro and macrovascular complications in diabetic patients[7, 8]. VWF/ADAMTS13 and ADAMTS13 activity/ADAMTS13 Ag ratio have also been described as markers of endothelial dysfunction [9] and a reduced activity of ADAMTS13 was shown to be associated with renal disease in patients with type 2 diabetes mellitus (DM2) [10].

Moreover, elevated plasma levels of D-Dimer have been found in DM1 patients with microvascular complications, and it seems that hypercoagulability might be involved with the progression of atherosclerosis as well as with renal dysfunction in diabetic patients [11, 12]. D-Dimer consists of an important biomarker of hypercoagulability, since it is a fibrin degradation product that derives only from fibrin, but not from fibrinogen, and therefore is specific to fibrinolytic activity secondary to fibrin formation [13].

Based on the evidence that these biomarkers are associated with endothelial dysfunction and hypercoagulability, this study aimed to investigate the relationship among VWF, ADAMTS13, and D-Dimer with different levels of renal dysfunction in DM1 patients. To the best of our knowledge, our study was the first one to evaluate ADAMTS13 Ag levels and ADAMTS13 activity in DM1 with different degrees of renal dysfunction. Since these associations are unknown, this study may provide relevant information as the first step in identifying the utility of these biomarkers to predict the progression of renal disease in DM1.

## Materials and Methods

### Ethics

All procedures performed in this study were in accordance with the ethical standards of the institutional and/or national research committee and with the 1964 Declaration of Helsinki and its later amendments or comparable ethical standards. This study was approved by the Research Ethics Committee of Federal University of Minas Gerais (CAAE– 0392.0.203.000–11) and written informed consent was obtained from all individual participants included in the study. The research protocol did not interfere with any medical recommendations or prescriptions.

### Studied population

The clinical records of all 240 DM1 patients receiving assistance at Endocrinology Ambulatories of the University Hospital (*Hospital das Clínicas*) and Santa Casa de Misericórdia/Belo Horizonte, Brazil, from November 2011 to September 2012, for biannual check up, were analysed. After application of exclusion criteria, which consisted on hepatic disease, alcoholism, hemostatic abnormalities, malignant diseases, acute infectious, pregnancy, renal hyperfiltration, undergoing hemodialysis and history of kidney transplantation or cardiovascular diseases, 15 patients were excluded from the study. Therefore, 125 patients with clinical and laboratorial diagnosis of DM1 [14], 18 to 60 years of age, were invited to participate of this study and all of them agreed to participate. Blood samples were drawn from these selected patients and biochemistry and haemostatic parameters were assessed. 16 patients presented hyperfiltration (eGFR ≥ 130 mL/min/1.73m^2^) and were also excluded from the study. Finally, 109 DM1 patients were included in this observational case-control study.

### Study protocol

A detailed history and clinical variables of each patient were obtained from medical records: age, sex, BMI, time of diabetes diagnosis, presence of diabetes complications such as retinopathy and neuropathy, and use of medication such as antihypertensive, statin, and acetylsalicylic acid (AAS).

DM1 patients were placed into three groups, according to level of renal function: patients with eGFR-MDRDa ≥ 90 and < 130 mL/min/1.73m^2^, n = 52 (control group); patients with eGFR-MDRDa ≥ 60 and < 90 mL/min/1.73m^2^, n = 29 (mild renal dysfunction group); and patients with eGFR-MDRDa < 60 mL/min/1.73m^2^, but not undergoing hemodialysis, n = 28 (severe renal dysfunction group) [14]. They were also placed into three groups according to urinary albumin excretion (UAE): normoalbuminuria, n = 53, microalbuminuria, n = 26, and proteinuria, n = 30 [15].

### Determination of biochemistry parameters

Fasting glucose and creatinine levels were determined by enzymatic methods in serum samples, and HbA1c was determined by the immunoturbidimetric method in EDTA whole blood samples, using dry chemistry technology kits. UAE was determined in urine samples collected after at least 4 hours of urinary retention in the morning, and urinary albumin was normalized by urinary creatinine. Urinary albumin was evaluated by the immunoturbidimetric method and urinary creatinine was assessed by the enzymatic method, using dry chemistry technology kits.

Normoalbuminuria was defined as < 30 mg/g of creatinine, microalbuminuria as ≥ 30 and < 300 mg/g of creatinine, and proteinuria as ≥ 300 mg/g of creatinine. The presence of microalbuminuria or proteinuria was confirmed in two out of three occasions over a period between three and six months [15].

### Estimation of glomerular filtration rate

The estimated glomerular filtration rate was calculated using the abbreviated Modification of Diet in Renal Disease formula [eGFR-MDRDa: 186 x plasma creatinine (mg/dL)^-1,154^ x age (years)^-0,203^ x 0.742 (if female) x 1.212 (if black)] [16].

### Hemostatic parameters measurement

VWF, ADAMTS13 antigen, and D-Dimer plasma levels were determined by ELISA. ADAMTS13 activity was assessed by fluorescence resonance energy transfer (FRET) assay. Intra- and inter-assay coefficients of variations were, respectively, 9% and 13% for VWF, 4.0% and 7.3% for ADAMYS13 antigen, <6% and <10% for D-Dimer, and 4.1% and 4.4% for ADAMTS13 activity.

### Statistical analysis

Statistical comparisons were performed using SPSS software (version 20.0, SPSS). The Shapiro-Wilk test was used to test whether continuous variables were normally distributed. Normally-distributed data were expressed as mean ± SD and compared by ANOVA and Student’s t-test. Not normally distributed data were expressed as median (percentiles 25%– 75%) and compared by the Kruskal-Wallis H test and Mann-Whitney U test, followed by Bonferroni correction. Categorical variables were expressed as frequencies and compared using the chi-square test (χ^2^). A bivariate logistic regression analysis was performed to assess which hemostatic variables were associated with eGFR-MDRDa < 90 mL/min/1.73m^2^, eGFR-MDRDa < 60 mL/min/1.73m^2^, UAE ≥ 30 mg/g of creatinine, and UAE ≥ 300 mg/g of creatinine. A multivariate logistic regression analysis was also performed to assess which variables were independently associated with the same dependent variables described above. Variables included in this analysis were previously associated with renal dysfunction and renal injury in bivariate logistic regression analysis (p < 0.2) and consisted on VWF, ADAMTS13 activity/ADAMTS13 Ag ratio, D-Dimer, age, BMI, time of diabetes diagnosis, HbA1c, use of antihypertensive, use of statin andAAS. Age, BMI, time of diabetes diagnosis and HbA1c were classified into different categories, using the following values as a cut off: < 7% and ≥ 7%, for HbA1c; ≥ 18 and < 30 years old, ≥ 30 and < 45 years old, ≥ 45 years old, for age; < 25 kg/m^2^, ≥ 25 and < 30 kg/m^2^, ≥ 30 kg/m^2^, for BMI; and ≤ 10 years, > 10 and ≤ 20 years, > 20 years, for time of diabetes diagnosis. Differences were considered significant when P≤0.05.

## Results

### Characteristics of the study group

Characteristics and clinical variables of the 109 DM1 patients included in this cross-sectional study are shown in Table 1.

**Table 1:** Characteristics of DM1 patients according to estimated glomerular filtration rate.

	Control group	Mild renal dysfunction group	Severe renal dysfunction group	p
**Number of Individuals (n)**	52	29	28	
**Age (years)**	32 (25–37)	32 (28–35)	41 (32–48)** ^†^	0.001**
				0.001^†^
**Sex/male (n, %)**	22 (56.4)	5 (12.8)	12 (30.8)	NS
**BMI (kg/m** ^**2**^ **)**	24.6 ± 3.7	23.7 ± 2.7	22.2 ± 2.4**	0.007**
**Time of Diagnosis (years)**	19 ± 8	19 ± 7	22 ± 5	NS
**Retinopathy (n, %)**	15 (29.4)	11 (21.6)	25 (49.0)** ^†^	< 0.001**
				< 0.001^†^
**Neuropathy (n, %)**	6 (31.6)	7 (36.8)	6 (31.6)	NS
**Use of Antihypertensive (n, %)**	26 (36.1)	19 (26.4)	27 (37.5)** ^†^	< 0.001**
				< 0.001^†^
**Use of Statin (n, %)**	8 (21.1)	12 (31.6)*	18 (47.4)**	< 0.001*
				< 0.001**
**Use of AAS (n, %)**	4 (20.0)	3 (15.0)	13 (65.0)** ^†^	< 0.001**
				< 0.001^†^
**Fasting Glucose (mg/dL)**	145 (92–253)	159 (91–217)	128 (81–280)	NS
**HbA1c (%)**	8.3 ± 1.2	8.0 ± 1.1	8.5 ± 1.3	NS
**Creatinine (mg/dL)**	0.74 (0,67–0.85)*	1.00 (0.88–1.10)*	1.66 (1.41–2.11)** ^†^	< 0.001*
				< 0.001**
				< 0.001^†^
**eGFR-MDRDa (mL/min/1.73m** ^**2**^ **)**	109 ± 11	74 ± 10*	38 ± 13** ^†^	< 0.001*
				< 0.001**
				< 0.001^†^
**UAE (mg/g of creatinine)**	5 (3–13)	28 (10–154)*	496 (64–1417)** ^†^	< 0.001*
				< 0.001**
				< 0.001^†^

Normally-distributed data were expressed as mean ± SD and compared by ANOVA and T test. Not normally distributed data were expressed as median (percentiles 25%– 75%) and compared by the Kruskal-Wallis H test and Mann-Whitney U test, followed by Bonferroni correction. Categorical variables were expressed as frequencies n (%) and compared using the chi-square test (χ^2^). Body mass index (BMI), time of diagnosis, HbA1c and glomerular filtration rate estimated by MDRDa formula (eGFR-MDRDa) were normally distributed. Age, fasting glucose, creatinine and urinary albumin excretion (UAE) were not normally distributed.

Control group: patients with eGFR-MDRDa ≥ 90 and < 130 mL/min/1.73m^2^.

Mild renal dysfunction group: patients with eGFR-MDRDa ≥ 60 and < 90 mL/min/1.73m^2^.

Severe renal dysfunction group: patients with eGFR-MDRDa < 60 mL/min/1.73m^2^.

* P < 0.05 for mild renal dysfunction group compared to control group.

** P < 0.05 for severe renal dysfunction group compared to control group.

^†^ P < 0.05 for severe renal dysfunction groupcompared to mild renal dysfunction group.

NS = not significant. AAS = acetylsalicylic acid

Patients with severe renal dysfunction were older than thosewith mild renal dysfunction and the control group (P = 0.001). Patients with severe renal dysfunction had lower BMI than that found in the control group (P = 0.007). There were no significant differences among the groups regarding sex, time of diabetes diagnosis, fasting glucose, HbA1c, and presence of neuropathy. However, a higher frequency of retinopathy was observed in the group of patients with severe renal dysfunction as compared to the other groups (P<0.001). There was also a higher frequency of use of antihypertensive and AAS in the severe renal dysfunction group of patients as compared to the other groups (P < 0.001) and the use of statin in patients with mild and severe renal dysfunction as compared to the control group (P < 0.001).

### VWF, ADAMTS13, and D-Dimer

Plasma levels of VWF, ADAMTS13 Ag, ADAMTS13 activity, and D-Dimer were measured in diabetic patients, after which VWF/ADAMTS13Ag, VWF/ADAMTS13 activity and ADAMTS13 activity/ADAMTS13 Ag ratios were calculated (Table 2).

**Table 2:** VWF, ADAMTS13Ag and D-Dimer plasma levels, ADAMTS13 activity, and ratios in DM1 patients classified according to estimated glomerular filtration rate.

	Control group	Mild renal dysfunction group	Severe renal dysfunction group	P
**Number of Individuals (n)**	52	29	28	
**VWF (mU/mL)**	1031 ± 264	1290 ± 377*	1396 ± 408**	0.001*
				< 0.001**
**ADAMTS13Ag (ng/mL)**	309 (250–528)	503 (286–603)*	549 (351–635)**	0.029*
				0.002**
**ADAMTS13 Activity (%)**	95 ± 16	104 ± 20	108 ± 19**	0.006**
**VWF/ ADAMTS13Ag**	2.9 ± 1.0	2.7 ± 1.0	2.7 ± 1.1	NS
**VWF/ ADAMTS13 Activity**	11.7 ± 4.0	12.8 ± 4.2	13.2 ± 4.6	NS
**ADAMTS13 Activity/ ADAMTS13Ag**	0.30 (0.19–0.39)	0.20 (0.16–0.30)*	0.19 (0.18–0.28)**	0.013*
				0.015**
**D-Dimer (ng/mL)**	178 (128–264)	239 (195–385)*	361 (232–536)** ^†^	0.006*
				< 0.001**
				0.019^†^

Normally-distributed data were expressed as mean ± SD and compared by ANOVA and T test. Not normally distributed data were expressed as median (percentiles 25%– 75%) and compared by the Kruskal-Wallis H test and Mann-Whitney U test, followed by Bonferroni correction. Von Willebrand factor (VWF), ADAMTS13 Activity, VWF/ADAMTS13Ag and VWF/ADAMTS13 Activity were normally distributed. ADAMTS13Ag, ADAMTS13 Activity/ADAMTS13Ag and D-Dimer were not normally distributed.

Control group: patients with eGFR-MDRDa ≥ 90 and < 130 mL/min/1.73m^2^.

Mild renal dysfunction group: patients with eGFR-MDRDa ≥ 60 and < 90 mL/min/1.73m^2^.

Severe renal dysfunction group: patients with eGFR-MDRDa < 60 mL/min/1.73m^2^.

* P < 0.05 for mild renal dysfunction group compared to control group.

** P < 0.05 for severe renal dysfunction group compared to control group.

^†^ P < 0.05 for severe renal dysfunction groupcompared to mild renal dysfunction group.

NS = not significant.

VWF plasma levels were elevated in patients with mild and severe renal dysfunction as compared to the control group (P = 0.001 and P < 0.001, respectively). ADAMTS13 Ag levels were also elevated in mild and severe renal dysfunction groups of patients as compared to the control group (P = 0.029 and P = 0.002, respectively), while ADAMTS13 activity was elevated only in severe renal dysfunction group as compared to the control group (P = 0.006).

No significant differences were observed among the groups regarding the VWF/ADAMTS13 Ag and VWF/ADAMTS13 activity ratios. Conversely, the ADAMTS13 activity/ADAMTS13 Ag ratio was reduced in patients with mild and severe renal dysfunction as compared to the control group (P = 0.013 and P = 0.015, respectively).

D-Dimer plasma levels were elevated in patients with mild and severe renal dysfunction as compared to the control group (P = 0.006 and P < 0.001, respectively) and was also higher in patients with severe renal dysfunction as compared to the mild renal dysfunction group of patients (P = 0.019).

Differences among VWF, ADAMTS13 Ag, ADAMTS13 activity, and D-Dimer were also evaluated in patients classified according to UAE to evaluate the association of these hemostatic parameters with the development of renal injury (Table 3). The association of these parameters with UAE was similar to that observed in renal dysfunction.

**Table 3:** VWF, ADAMTS13Ag and D-Dimer plasma levels, ADAMTS13 activity and ratios in the DM1 patients classified according to urinary albumin excretion.

	Patients with normoalbuminuria	Patients with microalbuminuria	Patients with macroalbuminuria	p
**Number of Individuals (n)**	53	26	30	
**VWF (mU/mL)**	1050 ± 280	1319 ± 377*	1428 ± 431**	0.003*
				< 0.001**
**ADAMTS13Ag (ng/mL)**	297 (235–507)	504 (384–609)*	571 (338–661)**	0.002*
				< 0.001**
**ADAMTS13 Activity (%)**	95 ± 17	100 ± 15	113 ± 21** ^†^	< 0.001**
				0.007^†^
**VWF/ ADAMTS13Ag**	3.3 (1.9–4.1)	2.9 (2.1–3.2)	2.4 (1.9–3.5)	NS
**VWF/ ADAMTS13 Activity**	11.3 ± 3.7	13.5 ± 4.0	11.6 ± 3.2	NS
**ADAMTS13 Activity/ ADAMTS13Ag**	0.32 (0.20–0.40)	0.18 (0.18–0.19)*	0.18 (0.18–0.20)**	0.002*
				< 0.001**
**D-Dimer (ng/mL)**	200 ± 73	292 ± 153*	441 ± 253** ^†^	0.001*
				< 0.001**
				0.013^†^

Normally distributed data were expressed as mean ± SD and compared by ANOVA and T test. Not normally distributed data were expressed as median (percentiles 25%– 75%) and compared by the Kruskal-Wallis H test and Mann-Whitney U test, followed by Bonferroni correction. Von Willebrand factor (VWF), ADAMTS13 Activity, VWF/ADAMTS13 Activity and D-Dimer were normally distributed. ADAMTS13Ag, VWF/ADAMTS13Ag and ADAMTS13 Activity/ADAMTS13Ag were not normally distributed.

* P < 0.05 for patients with microalbuminuria compared to patients with normoalbuminuria

** P < 0.05 for patients with proteinuria compared to patients with microalbuminuria

^†^ P < 0.05 for patients with proteinuria compared to patients with microalbuminuria.

NS = not significant.

### Bivariate and Multivariate logistic regression analysis

The bivariate logistic regression analysis demonstrated that VWF, ADAMTS13 Ag, ADAMTS13 activity, ADAMTS13 activity/ADAMTS13 Ag and D-Dimer were associated with eGFR-MDRDa < 90 mL/min/1.73m^2^, eGFR-MDRDa < 60 mL/min/1.73m^2^, UAE ≥ 30 mg/g of creatinine and UAE ≥ 300 mg/g of creatinine (Table 4).

**Table 4:** Association between hemostatic parameters and severe + mild renal dysfunction; severe renal dysfunction; macroalbuminuria + microalbuminuria; macroalbuminuria.

Variable	Severe renal dysfunction + Mild renal dysfunction x Control group*	Severe renal dysfunction x Mild renal dysfunction + Control group**	Macroalbuminuria + Microalbuminuria x Normoalbuminuria^†^	Macroalbuminuria x Microalbuminuria + Normoalbuminuria^††^
**VWF**	1.003	1.002	1.003	1.002
	(1.001–1.004)	(1.001–1.003)	(1.001–1.004)	(1.001–1.003)
	P < 0.001	P = 0.002	P < 0.001	P = 0.001
**ADAMTS13Ag**	1.003	1.003	1.005	1.004
	(1.001–1.004)	(1.001–1.005)	(1.003–1.007)	(1.001–1.006)
	P = 0.001	P = 0.016	P < 0.001	P = 0.002
**ADAMTS13 activity**	1.033	1.029	1.036	1.053
	(1.010–1.057)	(1.004–1.025)	(1.013–1.060)	(1.024–1.082)
	P = 0.005	P = 0.025	P = 0.002	P < 0.001
**ADAMTS13 Activity/ ADAMTS13Ag**	0.001	0.006	1 x 10^-4^	1 x 10^-4^
	(1 x 10^-4^–0.057)	(1 x 10^-4^–0.942)	(1 x 10^-4^–2 x 10^-4^)	(1 x 10^-4^–0.014)
	P = 0.001	P = 0.047	P < 0.001	P = 0.008
**D-Dimer**	1.009	1.007	1.009	1.007
	(1.004–1.013)	(1.004–1.011)	(1.004–1.013)	(1.003–1.010)
	P < 0.001	P < 0.001	P < 0.001	P < 0.001

Data was evaluated by bivariate logistic regression analysis and are presented as odds ratio (95% Confidence Interval). VWF = von Willbrand factor.

* P < 0.05 for severe renal dysfunction and mild renal dysfunction groups (eGFR-MDRDa < 90 mL/min/1.73m^2^) compared to control group (eGFR-MDRDa ≥ 90 mL/min/1.73m^2^).

** P < 0.05 for severe renal dysfunction group (eGFR-MDRDa < 60 mL/min/1.73m^2^) compared to mild renal dysfunction and control groups (eGFR-MDRDa ≥ 60 mL/min/1.73m^2^).

^†^ P < 0.05 for patients with macroalbuminuria and microalbuminuria (UAE ≥ 30 mg/g of creatinine) compared to patients with normoalbuminuria (UAE < 30 mg/g of creatinine).

^††^ P < 0.05 for patients with macroalbuminuria (UAE ≥ 300 mg/g of creatinine) compared to patients with microalbuminuria and normoalbuminuria (UAE < 300 mg/g of creatinine).

The multivariate logistic regression analysis showed that VWF, ADAMTS13 activity/ADAMTS13 Ag, D-Dimer, and use of statin were independently correlated with eGFR-MDRDa < 90 mL/min/1.73m^2^ when compared to eGFR-MDRDa ≥ 90 mL/min/1.73m^2^. Moreover, only D-Dimer and use of antihypertensive or AAS were independently correlated with eGFR-MDRDa < 60 mL/min/1.73m^2^ as compared to eGFR-MDRDa ≥ 60 mL/min/1.73m^2^. VWF, ADAMTS13 activity/ADAMTS13 Ag, and D-Dimer were also independently correlated with UAE ≥ 30 mg/g of creatinine when compared to UAE < 30 mg/g of creatinine. Furthermore, only D-Dimer and the use of AAS were independently correlated with UAE ≥ 300 mg/g of creatinine as compared to UAE < 300 mg/g of creatinine. Moreover, the multivariate logistic regression analysis has demonstrated that age, time of diabetes diagnosis, HbA1c and BMI were not independently correlated with eGFR-MDRDa < 90 or < 60 mL/min/1.73m^2^ and with UAE ≥ 30 or ≥ 300 mg/g (Table 5).

**Table 5:** Variables that correlated independently with severe + mild renal dysfunction; severe renal dysfunction; macroalbuminuria + microalbuminuria; macroalbuminuria.

Variable	Severe renal dysfunction + Mild renal dysfunction x Control group*	Severe renal dysfunction x Mild renal dysfunction + Control group**	Macroalbuminuria + Microalbuminuria x Normoalbuminuria^†^	Macroalbuminuria x Microalbuminuria + Normoalbuminuria^††^
**VWF**	1.003	NS	1.003	NS
	(1.001–1.005)		(1.001–1.005)	
	P = 0.008		P = 0.015	
**ADAMTS13 Activity/ ADAMTS13Ag**	1 x 10^-4^	NS	1 x 10^-10^	NS
	(3 x 10^-7^–0.109)		(1 x 10^-16^–1 x 10^-4^)	
	P = 0.008		P = 0.001	
**D-Dimer**	1.010	1.008	1.006	1.007
	(1.004–1.016)	(1.004–1.012)	(1.000–1.013)	(1.004–1.010)
	P = 0.002	P < 0.001	P = 0.047	P < 0.001
**Use of Antihypertensive**	NS	12.249	NS	NS
		(1.371–109.467)		
		P = 0.025		
**Use of Statin**	13.962	NS	NS	NS
	(3.191–61.083)			
	P < 0.001			
**Use of AAS**	NS	6.636	NS	4.794
		(1.711–25.735)		(1.449–15.863)
		P = 0.006		P = 0.010
**Age**	NS	NS	NS	NS
**Time of diabetes diagnosis**	NS	NS	NS	NS
**HbA1c**	NS	NS	NS	NS
**BMI**	NS	NS	NS	NS

Data was evaluated by multivariate logistic regression analysis and are presented as odds ratio (95% Confidence Interval). NS = not significant. BMI = body mass index. AAS = acetylsalicylic acid. VWF = von Willbrand factor).

* P < 0.05 for severe renal dysfunction and mild renal dysfunction groups (eGFR-MDRDa < 90 mL/min/1.73m^2^) compared to control group (eGFR-MDRDa ≥ 90 mL/min/1.73m^2^).

** P < 0.05 for severe renal dysfunction group (eGFR-MDRDa < 60 mL/min/1.73m^2^) compared to mild renal dysfunction and control groups (eGFR-MDRDa ≥ 60 mL/min/1.73m^2^).

^†^ P < 0.05 for patients with macroalbuminuria and microalbuminuria (UAE ≥ 30 mg/g of creatinine) compared to patients with normoalbuminuria (UAE < 30 mg/g of creatinine).

^††^ P < 0.05 for patients with macroalbuminuria (UAE ≥ 300 mg/g of creatinine) compared to patients with microalbuminuria and normoalbuminuria (UAE < 300 mg/g of creatinine).

## Discussion

There are scant data in the literature correlating the progression of nephropathy, endothelial dysfunction and hypercoagulability in DM1 patients. In this study, we used VWF, ADAMST13 and D-Dimer to assess endothelial dysfunction and hypercoagulability in DM1 patients with different and progressive levels of renal dysfunction.

High VWF levels were associated with mild and severe renal dysfunction, and with micro and macroalbuminuria, in the present study. Similar findings have been reported in both cross sectional [17] and longitudinal studies [18], in DM1 patients as well as in DM2 patients [19]. Moreover, there is evidence suggesting that rises in VWF levels precede the development of microalbuminuria in DM1 patients [20]. Altogether, these findings support the proposal that elevated VWF levels and endothelial dysfunction are associated with nephropathy in DM1, and might be useful to predict the development of renal disease in these patients.

The association between high ADAMTS13 levels with mild and severe renal dysfunction, and with micro and macroalbuminuria may be explained by the presence of a compensatory mechanism, by which ADAMTS13 synthesis is increased due to the marked elevation in VWF plasma levels, as nephropathy progresses. In agreement, this compensatory elevation in ADAMTS13 Ag levels and the increase in ADAMTS13 activity may be responsible for keeping VWF/ADAMTS13 Ag and VWF/ADAMTS13 activity ratios unchanged, as seen in patients with renal dysfunction. Conversely, a low ADAMTS13 activity/ADAMTS13 Ag ratio was associated with mild and severe renal dysfunction, and with micro and macroalbuminuria, suggesting that the rise in ADAMTS13 Ag levels is not accompanied by a proportional increase in ADAMTS13 activity and VWF cleavage in these patients. This imbalance between ADAMTS13 activity and ADAMTS13 Ag levels may well be associated with the progressive inflammatory process, frequently seen in DM1 [4]. Thishypothesis is supported by an *in vitro* study [21], which has demonstrated that IL-6 inhibited VWF cleavage by ADAMTS13. However, as DM1 is an autoimmune disease, auto-antibodies against ADAMTS13 could also partially explain the imbalance between ADAMTS13 activity and ADAMTS13 Ag levels, by inhibiting ADAMTS13 activity [22].

D-Dimer levels were also associated with mild and severe renal dysfunction, and with micro and macroalbuminuria. To our knowledge, a single study [11] has found elevated levels of D-Dimer in children and adolescents with DM1 and nephropathy. High D-Dimer plasma levels were also verified in DM2 patients with increased UAE [11,23]. Increased D-Dimer levels in patients with renal dysfunction may result from endothelial dysfunction, since release of VWF promotes platelet adhesion and aggregation and, thus, microthrombi formation [6]. Endothelial vascular damage still impairs the conversion of protein C into its activated form, since this activation depends on the endothelial protein C receptor and thrombomodulin, which are expressed at high levels in undamaged microvasculature [24]. Proteinuria may also explain the more pronounced elevation in D-Dimer levels in patients with severe renal dysfunction due to loss of important natural anticoagulant proteins, such as antithrombin, protein C and protein S, intensifying their hypercoagulability status [25].

According to the multivariate regression analysis, antihypertensive use was independently associated with severe renal dysfunction, as expected, given that renal dysfunction contributes to the development of hypertension and angiotensin converting enzyme inhibitors may protect renal function and prevent the progression of nephropathy [26]. The independent association of statin use with mild and severe renal dysfunction, and aspirin use with severe renal dysfunction, could also be expected, since GFR decline is a risk factor for dyslipidemia [26] and the use of an antiplatelet agent is usually recommended to patients presenting nephropathy [26], due to increased risk of cardiovascular disease [27, 28]. Interestingly, statin has pleiotrophic effects, and in addition of reducing cholesterol levels, it can also reduce inflammation, oxidative stress, platelet aggregation [29] and D-Dimer levels, in healthy subjects [30]. Therefore, it could be expected that D-Dimer levels would be even higher in patients with mild and severe renal dysfunction, if most of them were not taken statin. In fact, D-Dimer levels were significantly lower (287 ± 112 ng/mL) in patients with mild and severe renal dysfunction that were taking statin (n = 30), as compared with those not using statin (n = 27) (383 ± 209 ng/mL) (P = 0.043) (data not shown). Aspirin may also affect coagulation, by inhibiting platelet aggregation and consequently microthrombi formation [31] and lowering D-Dimer levels in this group of patients. In agreement, D-Dimer levels were significantly lower (283 ± 108 ng/mL) in patients with severe renal dysfunction that were taking aspirin (n = 13), as compared with those not using aspirin (n = 15) (434 ± 175 ng/mL) (P = 0.014) (data not shown). Therefore, the hypercoagulability status of patients with severe renal dysfunction could be even higher, since these medications may have impacted their D-Dimer levels. It is noteworthy that the patients included in this study did not have history of thrombosis or malignant diseases and other conditions that are frequently associated with hypercoagulability. Moreover, the single clinical characteristics significantly different among groups, BMI, time of diabetes diagnosis and age, were not independently associated with levels of coagulation markers, as indicated by the multivariate regression analysis. However, the more frequent use of these medications among patients with severe renal dysfunction did not prevent the disclosure of significant independent associations between different stages of renal dysfunction and hypercoagulability status.

There is a known relationship between the progression of nephropathy and an increased risk of cardiovascular disease in both DM1 and DM2 [32, 33]. Elevated levels of VWF and D-Dimer are also associated with the development of cardiovascular disease [34, 35]. Since this is not a prospective study, patients were not followed up for cardiovascular events. However, the results reported herein may guide future longitudinal studies aiming to evaluate the link between nephropathy, hypercoagulability biomarkers and cardiovascular disease. Since VWF, ADAMTS13 activity and D-Dimer levels are also elevated in DM2 patients with nephropathy, we believe that endothelial dysfunction and hypercoagulability may play an important role in the progression of renal dysfunction in both DM1 and DM2 [21, 23, 26].

Interestingly, our findings suggest that endothelial dysfunction and hypercoagulability are evident at early stages of renal disease, whilst hypercoagulability is also associated with later stages of renal dysfunction. Therefore, VWF, ADAMST13 and D-Dimer levels may complement each other on the follow-up of renal dysfunction in DM1 patients as well as their risk for cardiovascular disease and progression of renal dysfunction. Since these markers are also elevated in DM2 patients with nephropathy and endothelial dysfunction and hypercoagulability may also play an important role in DM2 [21, 23, 26], these associations may well be significant and extended to DM2. However, as previously mentioned, longitudinal studies are needed to confirm that these coagulation factors are useful markers of diabetic nephropathy.

In conclusion, elevated VWF, ADAMTS13 Ag, and D-Dimer plasma levels and increased ADAMTS13 activity, as well as decreased ADAMTS13 activity/ADAMTS13 Ag ratio, are associated with eGFR declining and UAE increasing in DM1 patients. The small sample size and cross sectional design of this study limited our analysis on the relationship between endothelial dysfunction and hypercoagulability with the progression of nephropathy. Despite the limitations of a cross sectional study, our data suggest that levels of endothelial dysfunction and hypercoagulability biomarkers are altered in DM1 patients with nephropathy, as evaluated by GFR decline or by increased UAE. These findings may be useful for guiding future longitudinal studies aimed at acquiring a better understanding of the mechanisms linking these aspects and nephropathy progression in DM1 patients. By means of longitudinal studies, it will be possible to identify the unity of these biomarkers are potential early biomarkers of disease progression, allowing for needed early intervention.
